# Reactive strategies for containing developing outbreaks of pandemic influenza

**DOI:** 10.1186/1471-2458-11-S1-S1

**Published:** 2011-02-25

**Authors:** Sigrún Andradóttir, Wenchi Chiu, David Goldsman, Mi Lim Lee, Kwok-Leung Tsui, Beate Sander, David N Fisman, Azhar Nizam

**Affiliations:** 1 H. Milton Stewart School of Industrial and Systems Engineering, Georgia Institute of Technology, Atlanta, Georgia, 30332, USA; 2 Toronto Health Economics and Technology Assessment Collaborative, Toronto, Ontario, M5S 3M2, Canada; 3 Department of Health Policy, Management and Evaluation, University of Toronto, Toronto, Ontario, M5T 3M6, Canada; 4 Division of Clinical Decision-Making and Health Care Research, University Health Network, Toronto, Ontario, M5G 2C4, Canada; 5 Department of Epidemiology, Dalla Lana School of Public Health, University of Toronto, Toronto, Ontario, M5T 3M7, Canada; 6 Department of Biostatistics and Bioinformatics, Emory University, Atlanta, Georgia, 30322, USA

## Abstract

**Background:**

In 2009 and the early part of 2010, the northern hemisphere had to cope with the first waves of the new influenza A (H1N1) pandemic. Despite high-profile vaccination campaigns in many countries, delays in administration of vaccination programs were common, and high vaccination coverage levels were not achieved. This experience suggests the need to explore the epidemiological and economic effectiveness of additional, reactive strategies for combating pandemic influenza.

**Methods:**

We use a stochastic model of pandemic influenza to investigate realistic strategies that can be used in reaction to developing outbreaks. The model is calibrated to documented illness attack rates and basic reproductive number (R_0_) estimates, and constructed to represent a typical mid-sized North American city.

**Results:**

Our model predicts an average illness attack rate of 34.1% in the absence of intervention, with total costs associated with morbidity and mortality of US$81 million for such a city. Attack rates and economic costs can be reduced to 5.4% and US$37 million, respectively, when low-coverage reactive vaccination and limited antiviral use are combined with practical, minimally disruptive social distancing strategies, including short-term, as-needed closure of individual schools, even when vaccine supply-chain-related delays occur. Results improve with increasing vaccination coverage and higher vaccine efficacy.

**Conclusions:**

Such combination strategies can be substantially more effective than vaccination alone from epidemiological and economic standpoints, and warrant strong consideration by public health authorities when reacting to future outbreaks of pandemic influenza.

## Background

In April, 2009, the World Health Organization (WHO) announced the emergence of a new influenza A (H1N1) virus, and on June 11, 2009, it declared that the world was at the start of a new influenza pandemic [1]. WHO reported more than 414,000 laboratory-confirmed cases of H1N1 [2] — a gross underestimate, as many countries simply stopped counting individual cases. The US Centers for Disease Control and Prevention reported widespread influenza activity in forty-six states, with influenza-like illness (ILI) activity in October 2009 higher than what is seen during the peak of many regular flu seasons; and further, “Almost all of the influenza viruses identified … are 2009 H1N1 influenza A viruses” [3]. Countries found themselves in the position of having to react to contain already developing Fall outbreaks of influenza due to the new pandemic strain, a position they are likely to find themselves in again if and when future waves of pandemic influenza occur.

Research has suggested that mass vaccination of 60–70% of the population prior to the start of the flu season could effectively contain outbreaks due to pandemic strains [4-7]; and the public health preparedness plans of most countries have, accordingly, emphasized vaccination intervention strategies. However, the recent experience with H1N1 suggests that high vaccination coverage levels are difficult to achieve. In the case of H1N1, vaccination programs in most northern hemisphere countries started only after the virus was widely circulating. Furthermore, in some countries, supplies of vaccine were limited [8], delivery and administration occurred over a period of several months [9,10], and there were reports of public skepticism regarding the necessity and safety of vaccination [11,12], all of which were strong indicators suggesting that high vaccination coverage would be difficult to achieve. While many institutions in the US and elsewhere strongly encouraged and, in some cases, required workers to be vaccinated against seasonal influenza in 2009, H1N1 vaccination guidelines were focused mostly on people in certain age and high-risk groups [13]. Delays, limited and untimely vaccination supplies, and public reluctance to be vaccinated are likely to reduce the effectiveness of vaccination campaigns [4,5].

The issues outlined above for the recent outbreak of H1N1 are likely to occur again in future outbreaks of pandemic influenza. In this paper, we explore the effectiveness of realistic reactive intervention strategies implemented after the beginning of outbreaks of pandemic influenza. We calibrate our model based on data for the H1N1 pandemic (see Tuite et al. [14]), and we investigate the impacts of (i) the moderate vaccination coverage levels which, based on past experience, are likely to be realized, as well as high levels which would be more ideal; (ii) very limited treatment of cases with antivirals and prophylaxis of cases’ households with antivirals; and (iii) limited and practical social distancing measures such as five-day closure of individual schools on an as-needed basis, encouragement of liberal leave policies in the workplace, and encouragement of self-isolation. Intervention strategies that combine these approaches are also studied (cf. Halloran et al. [15]). For all intervention strategies, we provide cost estimates associated with morbidity and mortality that take into account direct medical costs as well as economic consequences resulting from school closures and work loss.

## Methods

### The simulation model

**Figure 1:**
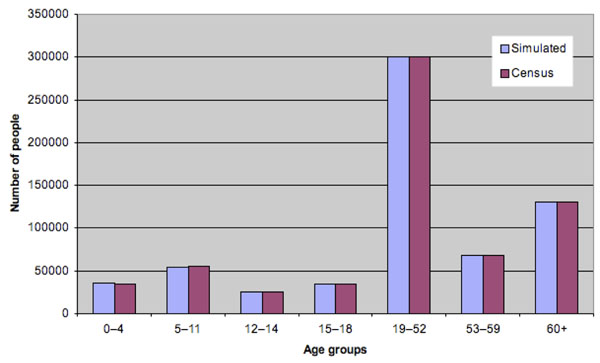
Age distribution for simulated population

**Figure 2:**
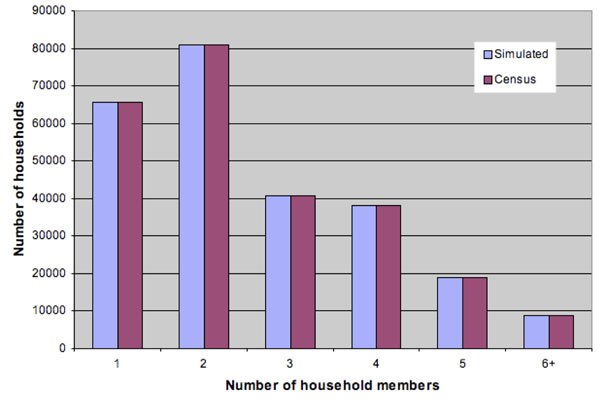
Household size distribution for simulated population

We developed a portable and adaptable stochastic, individual-level simulation model of influenza spread within a structured population. The simulator is similar to models developed by Longini et al. [7,16]. The simulation population of 649,565 people was generated stochastically to represent a typical North American city, namely, Hamilton (Ontario), Canada, which was chosen due to availability of demographic and epidemiological data necessary for constructing and calibrating the simulator. Our population is a collection of heterogeneous individuals with various attributes that impact whom they interact with (and hence whom they may infect or get infected by). More specifically, each individual has the following stochastically generated attributes: age, household, playgroup or daycare attended (for pre-school children), school attended (for school-age children), workgroup (for working adults), household census tract and workplace census subdivision, community, and neighborhood. As in [16], a community consists of approximately 2000 people living within the same census tract, and a neighborhood consists of approximately 500 people living within proximity to each other within the same community; also see the recent papers [17] and [18], which incorporate more-detailed individual-level behavior involving larger populations. Age and household-size distributions, shown in Figures 1 and 2, were matched to 2001 Canadian census data [19,20]. Household census tract assignments were made so that census tract population sizes were consistent with 2006 census statistics [21]. Workgroups were formed to match 2006 employment statistics [22] as well as census statistics on the geographical distribution of workers [23]. Rather than representing entire workplace institutions, we formed workgroups of size 20 to represent the typical number of co-workers an individual is likely to have close contact with during the day. Average playgroup, daycare, and lower and upper secondary school (i.e., middle and high school) contact group sizes were chosen for similar reasons; see the Appendix.

Susceptible people are assumed to have daily contacts with other individuals in their contact groups, i.e., their household and school or workgroups, as well as with people in their neighborhood and community. Infection of susceptibles depends on the number of infected persons in their contact groups, on the vaccine and antiviral-use status of susceptibles and their infectious contacts, and on age- and contact-group-specific per-contact transmission probabilities (Table 1). This disease transmission model is based on previously described models [7,16], and is detailed in the Appendix. People infected with influenza first pass through a latent / incubation period, during which they do not have influenza symptoms. They are not infectious until the last day of the period; at that point, they become half as infectious as if they were to develop symptoms in the subsequent period. During that subsequent infectious period, 67% will develop influenza symptoms and 33% will be asymptomatic (and will be half as infectious as those who are symptomatic) [7]. The model allows for people to withdraw from all of their mixing groups, except the household, if they become infected or have an infected child.

**Table 1:** Per-contact influenza infection transmission probabilities within contact groups

Contact Group	Transmission Probability
**Household**^1^	
Child-to-Child	0.8
Child-to-Adult	0.3
Adult-to-Child	0.3
Adult-to-Adult	0.4
**Community**^2^	
Pre-schooler	0.000005
School child	0.000005
Adult (ages 19–52)	0.000075
Adult (ages 53+)	0.000055
**Daycares/Playgroups**^2^	
Daycares	0.028
Playgroups	0.018
**Schools**^2^	
Elementary schools	0.012
Middle schools	0.011
High schools	0.010
**Workgroups**	0.010

1. Within households, the probability that a symptomatic child (age 18 years or less) infects a susceptible child is 0.8; that a symptomatic child infects a susceptible adult (at least 19 years old), or that a symptomatic adult infects a susceptible child, is 0.3; that a symptomatic adult infects a susceptible adult is 0.4 [16].

2. Probability that a susceptible person in the age or school group is infected through contact with a symptomatic person in the group.

The simulator is calibrated to match documented illness attack rates and basic reproductive numbers (R_0_). Baseline (no-intervention) scenario age-group-specific attack rates were derived using 2009 estimates for the H1N1 basic reproductive number in Ontario [14,24,25] (see Table 2). These rates take into account reduced risk in adults born prior to 1957 [24]. A compartmental model parameterized in this way was well-calibrated to observed attack rates during the Fall pandemic wave in Ontario [25]. The simulator’s R_0_ value of 1.4 is also consistent with other published reports [4,26,27].

**Table 2:** Age-group-specific H1N1 influenza illness attack rates in Ontario, Canada, 2009, and calibrated attack rates

	Simulated Ontario Illness Attack Rates by the Percentage of Adults 53+ Years Old with Pre-existing Immunity^1^				Calibrated Attack Rates (AR)	
Age	30%	50%	70%		Age	AR

0–4	30.6%	31.0%	30.8%		0–4	29.5%
5–13	53.8%	55.0%	55.2%		5–18	55.9%
14–17	56.0%	57.1%	57.3%					
18–22	48.9%	49.7%	49.7%		19–52	40.8%
23–52	39.6%	39.8%	39.3%					
53–64	21.7%	15.3%	8.8%		53–59	14.3%
65+	19.1%	13.2%	7.5%		60+	11.0%
Overall	36.8%	35.4%	33.5%		Overall	34.1%

1. See the discussion in [25].

### Intervention strategies

We modeled a baseline case where no intervention takes place, along with strategies representing various combinations of vaccination, antiviral treatment and household prophylaxis, school closure, and general social distancing (see the results in Tables 3 and 4 and Supplementary Data Table S1 provided in “Additional File 1”). Each component of the strategies is described in detail below. Interventions are triggered in a particular simulation run when the overall illness attack rate reaches 0.01%. Twenty runs of the simulator were performed for each intervention strategy, from which average illness attack rates were calculated. We briefly describe the interventions under consideration.

#### Vaccination

We model both pre-vaccination as well as reactive strategies, with reactive vaccination programs beginning immediately, 30 days, or 60 days after the trigger. The delays model disruptions in vaccine production and supply chains. We allow enough doses to cover either 35% or 70% of the population. In reactive strategies, we consider cases where (i) all vaccines become available at the same time, and (ii) the doses become available in three equal-sized batches, two weeks apart, due to additional production and supply-chain disruptions. We study a low-efficacy single-dose vaccine (efficacy against susceptibility to infection, VEs = 0.3, and efficacy against infectiousness, VEi = 0.2) as well as a moderate-efficacy vaccine (VEs = 0.4, VEi = 0.5) [28]. Vaccine efficacy refers to the reduction, after vaccination, in the probability of becoming infected due to contact with an infected person (VEs), or to the reduction, after vaccination, in the probability of infecting a susceptible contact (VEi). Vaccine efficacy does not refer to the fraction of individuals having an immunogenic response to the vaccine (which is typically much larger than our measures).

Each day, our model randomly vaccinates any remaining unvaccinated individuals who are either uninfected or in the latent or asymptomatic phases of infection, all with equal probability based on the number of available doses. Moreover, protection from the vaccine builds over time, with 50% of the vaccine’s efficacy realized upon vaccination, and full protection after two weeks.

#### Antiviral treatment and household prophylaxis

We investigate strategies involving treatment of infected individuals with a five-day course of antivirals, as well as strategies that also allow for ten-day prophylaxis of the infected individuals’ household members. We assume that 1% of individuals do not complete their course**.** We use an antiviral efficacy against susceptibility (AVEs) of 0.3 and against infectiousness (AVEi) of 0.7 [16]. Individuals receive direct benefit from antivirals only while they are taking them. Antiviral use is considered alone and in combination with other intervention strategies. It is assumed that antiviral courses are available for 10% of the population and that they are distributed to infected individuals and their household members until the supply is exhausted.

#### School closure and social distancing

**Figure 3:**
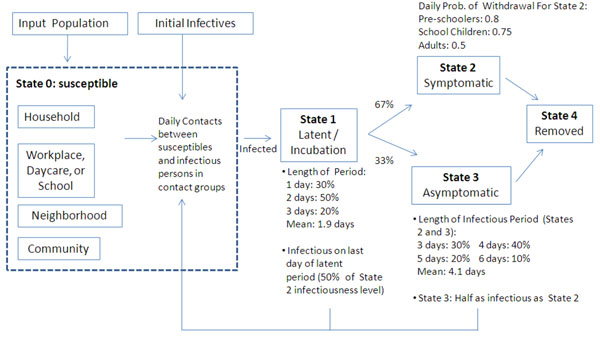
Simulation flowchart and modeled influenza natural history

We implement a rolling school closure model, where a daycare or school closes for five days if five or more cases are identified in that group. Given that infected individuals are on average infectious for 4.1 days (see Figure 3), closing schools for fewer than 5 days is unlikely to be very effective. It is possible for these groups to close more than once during the simulation. We also model a reduction in workplace and general community contacts of 20% (i.e., 20% of infected individuals in each contact group will not infect other members of the group). This represents the exercise of a general level of caution, including a modest limitation of contacts within workgroups (e.g., by invoking occasional telecommuting and other self-limiting behaviors, holding fewer large meetings, etc.) and also within the general community (e.g., reduction in attendance in social groups and larger community events, etc.).

### Economic cost estimation

We determine economic costs associated with the influenza outbreaks and modeled intervention strategies using methods described by Meltzer et al. [29]. We include medical spending due to illness, costs of antivirals and vaccines, and costs associated with teachers and other working adults staying home due to their own illness, illness of dependent children, or due to school closure. Medical spending includes co-payments and net payments for outpatient visits and hospitalization, as well as prescription and over-the-counter medications for influenza and complications or secondary infections. Costs are stratified by age-group and by low- or high-risk status of individuals with respect to complications of influenza. We also include the present value of earnings lost due to premature mortality.

Cost estimates and probabilities of risk status and of complications and death were taken from Meltzer et al. [29], with costs inflated using 2008 consumer price index and medical price index estimates [30-33]. These costs are combined with the data on age-specific attack rates, utilized vaccination doses, and days of school closure obtained from our simulation model. Details of the cost calculations are given in the Appendix.

## Results

With no intervention, the average overall illness attack rate is 34.1%, with an estimated total cost of $81.1 million (Table 3). Pre-vaccination of 35% of the population with a low-efficacy vaccine reduces the average overall illness attack rate to 26.1% (total cost $71.1 million), and with a moderate-efficacy vaccine to 18.8% (total cost $53.7 million). Not surprisingly, pre-vaccination of 70% of the population is more effective (overall average illness attack rate 12.0%, total cost $47.0 million for a low-efficacy vaccine; and 0.2% and $19.3 million with a moderate-efficacy vaccine; see Table 4).

**Table 3:** Average overall illness attack rates and total costs of interventions with 35% vaccination coverage

Intervention^1^	Delay in Initiation of Vaccination^2^	No Post-initiation Vaccination Delays		Post-initiation Vaccination Delays^3^	
		Attack Rate (%)	Cost (US$m)	Attack Rate (%)	Cost (US$m)

None		34.1	81.1		
A		31.3	75.9		
S		24.0	125.0		
A+S		9.2	48.0		
V_L_	Pre-vaccination	26.1	71.1		
V_L_	Reactive, no delay	28.8	77.7	28.8	77.7
V_L_	30-day delay	29.0	78.1	29.5	79.3
V_L_	60-day delay	30.7	82.2	32.2	86.0
V_M_	Pre-vaccination	18.8	53.7		
V_M_	Reactive, no delay	22.6	62.8	22.8	63.1
V_M_	30-day delay	23.0	63.7	24.6	67.5
V_M_	60-day delay	27.3	74.1	30.8	82.5
V_L_+A	Pre-vaccination	19.3	56.4		
V_L_+A	Reactive, no delay	25.2	70.6	25.3	70.8
V_L_+A	30-day delay	25.4	71.1	25.7	71.8
V_L_+A	60-day delay	26.2	72.9	27.1	75.0
V_M_+A	Pre-vaccination	2.1	16.1		
V_M_+A	Reactive, no delay	8.1	30.1	10.0	34.3
V_M_+A	30-day delay	12.4	40.2	15.8	48.2
V_M_+A	60-day delay	18.6	54.7	20.8	60.1
V_L_+S	Pre-vaccination	12.7	69.9		
V_L_+S	Reactive, no delay	17.3	93.6	17.5	95.7
V_L_+S	30-day delay	17.8	96.5	18.3	99.0
V_L_+S	60-day delay	18.6	101.9	19.6	108.8
V_M_+S	Pre-vaccination	2.3	19.6		
V_M_+S	Reactive, no delay	6.8	41.6	8.5	49.4
V_M_+S	30-day delay	9.9	56.3	15.4	87.3
V_M_+S	60-day delay	13.4	74.7	17.9	95.7
V_L_+A+S	Pre-vaccination	1.0	15.9		
V_L_+A+S	Reactive, no delay	3.9	29.2	4.5	32.2
V_L_+A+S	30-day delay	4.6	32.6	4.9	34.2
V_L_+A+S	60-day delay	4.8	33.8	5.4	36.8
V_M_+A+S	Pre-vaccination	0.2	11.9		
V_M_+A+S	Reactive, no delay	0.5	13.1	0.8	14.9
V_M_+A+S	30-day delay	1.2	16.6	1.6	18.6
V_M_+A+S	60-day delay	2.0	20.2	2.4	22.0

1. Abbreviations for modeled interventions: V (vaccination of up to 35% of the population), L (low efficacy), M (moderate efficacy), A (antiviral treatment and household prophylaxis of up to 10% of the population), S (school closure and social distancing).

2. Initial supply-chain delays which prevent immediate initiation of vaccination programs after the intervention trigger occurs.

3. Additional supply-chain delays, after initiation of the vaccination program, as a result of which vaccines become available in three equal batches, spaced two weeks apart.

**Table 4:** Average overall illness attack rates and total costs of interventions with 70% vaccination coverage

Intervention^1^	Delay in Initiation of Vaccination^2^	No Post-initiation Vaccination Delays		Post-initiation Vaccination Delays^3^	
		Attack Rate (%)	Cost (US$m)	Attack Rate (%)	Cost (US$m)

V_L_	Pre-vaccination	12.0	47.0		
V_L_	Reactive, no delay	22.2	71.1	22.4	71.6
V_L_	30-day delay	22.7	72.4	24.1	75.7
V_L_	60-day delay	27.1	83.0	30.4	89.4
V_M_	Pre-vaccination	0.2	19.3		
V_M_	Reactive, no delay	2.2	25.6	4.6	29.7
V_M_	30-day delay	8.1	39.5	13.3	50.2
V_M_	60-day delay	22.6	74.0	27.6	83.0
V_L_+A	Pre-vaccination	3.3	28.3		
V_L_+A	Reactive, no delay	17.3	61.1	17.7	62.0
V_L_+A	30-day delay	17.9	62.5	18.4	63.9
V_L_+A	60-day delay	19.9	67.4	22.0	72.4
V_M_+A	Pre-vaccination	0.1	20.7		
V_M_+A	Reactive, no delay	0.6	22.0	1.2	23.3
V_M_+A	30-day delay	2.4	26.2	4.4	30.9
V_M_+A	60-day delay	6.6	36.1	12.2	49.1
V_L_+S	Pre-vaccination	0.7	22.0		
V_L_+S	Reactive, no delay	5.9	46.0	7.5	53.1
V_L_+S	30-day delay	9.5	63.0	11.0	70.6
V_L_+S	60-day delay	13.3	82.6	15.4	96.6
V_M_+S	Pre-vaccination	0.04	19.1		
V_M_+S	Reactive, no delay	0.2	19.7	0.7	22.0
V_M_+S	30-day delay	1.5	25.9	3.2	34.7
V_M_+S	60-day delay	6.4	51.2	9.8	69.1
V_L_+A+S	Pre-vaccination	0.2	21.3		
V_L_+A+S	Reactive, no delay	1.8	28.4	2.6	32.0
V_L_+A+S	30-day delay	2.9	33.6	3.2	35.2
V_L_+A+S	60-day delay	3.8	37.8	4.6	41.7
V_M_+A+S	Pre-vaccination	0.02	20.6		
V_M_+A+S	Reactive, no delay	0.1	20.1	0.2	21.6
V_M_+A+S	30-day delay	0.5	22.8	0.7	23.8
V_M_+A+S	60-day delay	1.2	26.1	1.4	27.4

1. Abbreviations for modeled interventions: V (vaccination of up to 70% of the population), L (low efficacy), M (moderate efficacy), A (antiviral treatment and household prophylaxis of up to 10% of the population), S (school closure and social distancing).

2. Initial supply-chain delays which prevent immediate initiation of vaccination programs after the intervention trigger occurs.

3. Additional supply-chain delays, after initiation of the vaccination program, as a result of which vaccines become available in three equal batches, spaced two weeks apart

**Figure 4:**
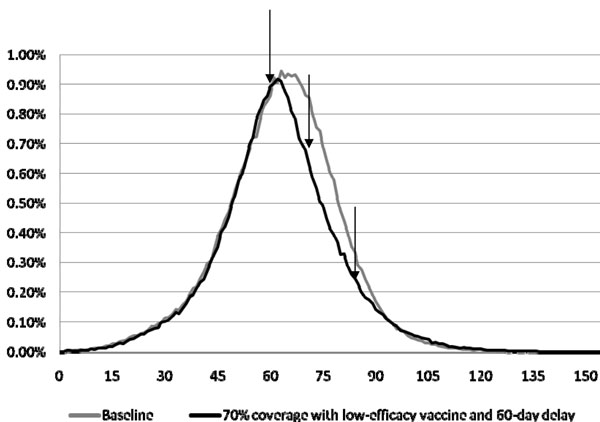
**Daily attack rates for (i) the case of 70% coverage of low-efficacy vaccine with 60-day initial delay, and (ii) the baseline case.** For case (i), the vaccine is given on the 60^th^ day followed by receipt of vaccine after two additional two-week delays (see arrows). Note that vaccine given on the 60^th^ day decreases the attack rate compared to the baseline; but the two subsequent receipts of vaccine do not result in additional benefits.

Reactive vaccination alone, of 35% of the population with a low-efficacy vaccine delivered in three batches, reduces the overall average illness attack rate to 28.8% (or 22.8% with a moderate-efficacy vaccine), with a total cost of $77.7 million ($63.1 million with a moderate-efficacy vaccine). Thirty- and 60-day delays in initiation of reactive vaccination, with vaccines delivered in three batches, result in attack rates of 29.5% (total cost $79.3 million) and 32.2% (total cost $86.0 million), respectively, for a low-efficacy vaccine, and 24.6% (total cost $67.5 million) and 30.8% (total cost $82.5 million), respectively, for a moderate-efficacy vaccine. Clearly, with a 60-day delay, interventions occur too late in the epidemic to have any meaningful effect (see Figure 4).

Antiviral use at low (10%) coverage alone results in an overall attack rate of 31.3% (total cost $75.9 million). School closure and social distancing alone result in an attack rate of 24.0%, with a total cost of $125.0 million.

Suppose we combine reactive low-efficacy vaccination of 35% of the population delivered in three batches, antivirals (10% coverage), and school closure and social distancing. Then the overall average illness attack rate is 4.5% (total cost $32.2 million) if no delays occur in the initiation of vaccination, and 5.4% (total cost $36.8 million) if a 60-day delay occurs. With a moderate-efficacy vaccine, the attack rate for this last scenario reduces to 2.4% (total cost $22.0 million). Similar relationships between interventions are apparent for interventions with 70% vaccination coverage, shown in Table 4. Vaccination coverage of 70% with a moderate-efficacy vaccine, combined with antiviral treatment and school closure, is highly effective, even with an initial 60-day delay and additional supply-chain disruptions (average illness attack rate 1.4%, total cost $27.4 million).

**Figure 5:**
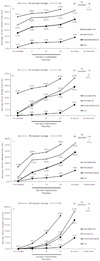
**Average overall illness attack rates (%) for modeled interventions**. Average overall illness attack rates for the following scenarios: no intervention; pre-vaccination; reactive vaccination with delays in initiation of 0, 30, and 60 days after the intervention trigger of a 0.01% overall illness attack rate; antiviral treatment or household prophylaxis with 10% population coverage (intervention “A”); rolling, as-needed five-day individual school closures and social distancing (20% reduction in workgroup and general community contacts—intervention “S”); antiviral use plus vaccination; school closure, and social distancing plus vaccination; antiviral use, school closure and social distancing, plus vaccination (“A+S”). Vaccination coverage is 35% of the population in Figures 5A and 5B; it is 70% of the population in Figures 5C and 5D. In reactive vaccination scenarios, additional supply-chain disruptions are assumed, such that vaccines are available in three equal batches, spaced two weeks apart, after initiation of vaccination programs. In Figures 5A and 5C, a low-efficacy vaccine is assumed (efficacy against susceptibility, VEs, 0.3; efficacy against infectiousness, VEi, 0.2). In Figures 5B and 5D, a moderate-efficacy vaccine is assumed (VEs, 0.5; VEi, 0.5).

We note that the results when all vaccines are available at the same time are better than those involving delivery in batches, and sometimes significantly so, especially for a moderate-efficacy vaccine (Tables 3 and 4). Figures 5A through 5D illustrate the comparative illness attack rates of the various intervention strategies discussed above for all combinations of low/moderate-efficacy vaccine delivered in three batches and at 35% / 70% coverage as a function of the initial delay in vaccination implementation due to supply-chain disruptions. The impact of vaccinating 70% of the population, rather than 35%, ranges from moderate to substantial, with the increased coverage being most beneficial when the vaccine is delivered in a timely manner, and the vaccine is either of moderate efficacy or of low efficacy applied in combination with other intervention strategies.

Complete (age-stratified and overall) average illness attack results for all modeled interventions are given in Supplementary Data Table S1. The comparative effectiveness of interventions is similar when age-group-specific results are studied.

**Figure 6:**
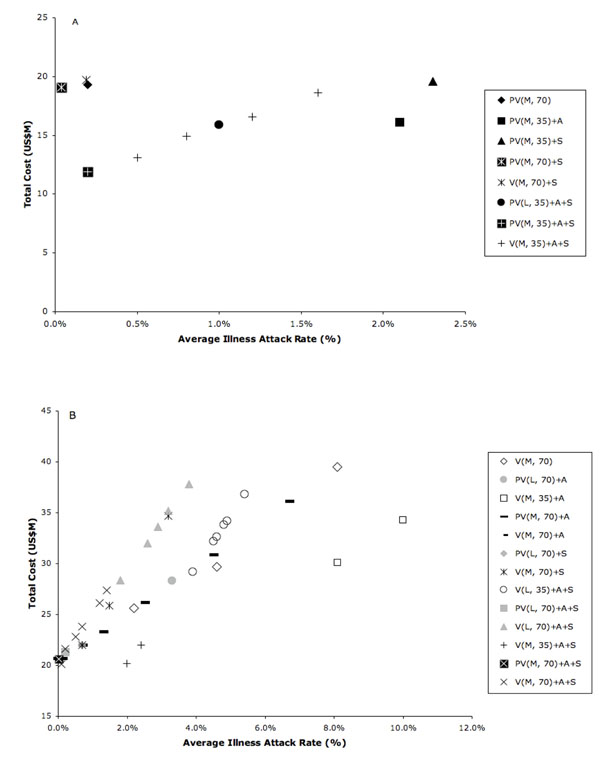
**Total cost of modeled intervention strategies (US$m) vs. average illness attack rate (%)** Figure 6A shows results for interventions with cost reductions of more than 75% compared with no intervention, and Figure 6B shows results for interventions with cost reductions of 50%−75% compared with no intervention. Abbreviations for modeled interventions: PV (pre-vaccination), V (vaccination), L (low-efficacy), M (moderate efficacy), 35 (35% coverage of population), 70 (70% coverage), A (antiviral treatment and household prophylaxis of up to 10% of the population), S (school closure and social distancing). Multiple occurrences of each plotting symbol may occur; occurrences at higher costs and illness attack rates represent interventions with longer supply-chain delays.

Figure 6A illustrates attack rate and total cost combinations for interventions that result in at least a 75% reduction in cost compared to no intervention. The closer to the origin, the more desirable an intervention is in terms of total cost and average illness attack rate. Aside from pre-vaccination strategies, we see that 70% reactive vaccination with a moderate-efficacy vaccine and school closure and social distancing, or even 35% reactive vaccination with a moderate-efficacy vaccine, antiviral use, and school closure, also result in substantial reductions in cost and attack rates. Figure 6B illustrates attack rate and cost results for interventions that result in more-modest 50%–75% reductions in cost compared to no intervention. Once again, several strategies combining vaccination, antiviral use, and school closure/social distancing are competitive with pre-vaccination.

## Discussion

Previously published research has shown that pre-vaccination of 60%–70% of the population can contain seasonal as well as pandemic influenza, but that delays in vaccination can greatly reduce the effectiveness of the vaccination programs [4-7]. Our model confirms these results for moderate-efficacy vaccines (Tables 3, 4, and S1). However, vaccination efforts in countries such as the US, Canada, and others began well after the first waves of H1N1 activity, and it is reasonable to believe that the same will be true in future outbreaks of pandemic influenza. In particular, in the event of an outbreak, it will likely take time to achieve high levels of vaccination coverage, and, if past experience with seasonal influenza vaccination campaigns is an indication, it is plausible that only low or moderate coverage will eventually be achieved. The results of our simulation model show that delayed and low-coverage reactive vaccination strategies (with a low-efficacy vaccine, plus limited use of antivirals) will not be enough to mitigate the pandemic or to significantly reduce total costs associated with influenza morbidity and mortality (based on results from Table 3, average illness attack rates are only reduced by 26% and total costs by 13%, compared to no intervention).

According to our model, combining rolling, limited-duration, as-needed closures of individual schools and a practical social distancing policy with 35% reactive low-efficacy vaccination coverage and low-level (10%) antiviral use can reduce illness attack rates by 89% compared to no intervention, as well as total costs by 64%. Similarly, combining interventions in this manner reduces overall attack rates by 99% and costs by 84% when a moderate-efficacy vaccine is available. This strategy remains highly effective even when delays in implementing vaccination of up to 60 days occur. Previously published results have left open the question of how costly interventions involving school closure might be [5]. Our results show that reactive combination strategies that include practical school closure measures, when diligently implemented, can reduce total costs associated with influenza morbidity and mortality substantially.

Our model has several limitations. We do not consider vaccination strategies targeted to high-risk groups, which could reduce costs associated with complications from influenza. We have not modeled co-circulating strains of seasonal and pandemic influenza or possible resistance to antiviral drugs (although, to mitigate this limitation, our model assumes only low coverage with antivirals, as well as interventions without antivirals). As is always the case with simulation models, continuing follow-up analyses are needed, including: (i) sensitivity to model parameters; (ii) sensitivity to model intervention triggers (e.g., overall illness attack rate, numbers of cases detected in schools, etc.); (iii) sensitivity to R_0_, which can be heterogeneous across cities and countries; and (iv) results for new H1N1 natural history and transmission parameters, and new cost estimates for complications resulting from H1N1 illness, as they become known.

Our model has several strengths. We model a large, realistic, heterogeneous population, base the simulation model on well-studied and documented stochastic simulators, calibrate to actual H1N1 attack rates and most-likely R_0_ values, and have the ability to model large numbers of scenarios in a relatively short amount of time on a desktop platform. The model also provides cost estimates that are useful for making policy decisions about potentially expensive interventions. In particular, we model and analyze a variety of interventions and combinations of interventions in terms of costs and efficacy. We also take into consideration reactive strategies incorporating supply-chain delays, and we identify strategies that effectively contain outbreaks and costs even in the presence of supply-chain delays, low vaccine efficacy, and low vaccine coverage.

## Conclusions

Our model illustrates the epidemiological effectiveness of a combination strategy involving short-term closures of individual schools on an as-needed basis, other practical social distancing activities, reactive vaccination of 35% or more of the population, and limited use of antivirals for treatment and prophylaxis. The model also quantifies the cost savings for this and alternative reactive strategies. Public health authorities should consider placing renewed emphasis on such combination strategies when reacting to possible additional waves of the current pandemic, or to new waves of future pandemics.

## Appendix

In this Appendix, we provide details on the simulation model as well as economic cost considerations.

### Simulation model

Our simulator is similar to those developed by Longini et al. for high-end computing platforms [7,16]; our simulator is programmed in C++ and runs on desktop platforms. Population structure and influenza transmission model details are given below.

#### Population structure

As discussed in the main text, the stochastically generated attributes for each person in our population of 649,565 included: age, household, playgroup or daycare attended (for pre-school children), school attended (for children 5–18 years of age), workgroup (for working adults and working 16–18 year old children), household census tract and workplace census subdivision, community (approximately 2000 people), and neighborhood (approximately 500 people). Thus individuals belong to three or four contact groups. In particular, each individual belongs to a household, neighborhood, and community. In addition, children younger than 16 belong to either a playgroup, daycare, or school, depending on age; most children in age range 16–18 belong to a school or workgroup; and most adults in age range 19–59 belong to a workgroup. Preschool children were categorized as belonging to a playgroup / daycare, each with 50% probability. We separated secondary schools into middle schools and high schools based on grade to allow different contact group sizes and to make our model more representative of mid-sized US cities. The numbers of playgroups, daycares, elementary, middle, and high schools in each community were based on Longini et al. [16], and were combined with the number of individuals in each category in our simulation population to obtain the contact group sizes. The number of working adults (19–59 years old) was based on census data [23]; and the number of working children (16–18 years old) was based on Ontario data on drop-out rates [34] and the employment rate for ages 15–24 [23].

#### Influenza transmission model

The simulator models influenza transmission over a 180-day period, within the contact groups previously defined. Figure 3 depicts a flowchart of the model. The modeled natural history and simulator dynamics parameters, described below and shown in Figure 3, were based on Longini et al. [7,19].

To initiate influenza outbreaks, simulations are seeded with approximately 100 randomly selected initial infectives, with all other individuals considered susceptible (state 0). Susceptible people have the opportunity, each day, to become infected in their contact groups. As discussed in the main text, the daily probability of infection for each susceptible person is determined by the number of infectious contacts in his contact groups, and on the per-contact probability of transmission for each type of contact. For example, the probability of a susceptible child who attends daycare being infected on a particular day is:

1 – [Pr(child is not infected in the household)

× Pr(child is not infected in the neighborhood)

× Pr(child is not infected in the community)

× Pr(child is not infected at the daycare center)].

Within each contact group, the probability of infection of a susceptible individual depends on the number of infectious individuals in the group. For example, suppose that *k1* children and *k2* adults in a household are infectious on a particular day. Then the probability of a susceptible household member being infected in that household on that day is:

1 – [Pr(not infected by a particular infected child in the household)*^k1^*

× Pr(not infected by a particular infected adult in the household)*^k2^*].

The number of infectious people in the contact groups (e.g., *k1* and *k2*), are random variables that are updated at the beginning of each day.

Age- and contact-group-specific per-contact probabilities of transmission of infection are given in Table 1. The probability that infection is transmitted from an infected person to a susceptible person also depends on whether the infectious person is symptomatic or asymptomatic. Table 1 shows the rates for symptomatic individuals. The transmission rates for asymptomatic individuals are half of those shown in Table 1. These probabilities are based on Longini et al. [7,16], with adjustments made to calibrate baseline (no intervention) results to age-group-specific illness attack rates and R_0_ estimates for novel A (H1N1) in Ontario [14,24,25]; see Table 2.

Once infected, people enter a 1–3 day latent period (state 1; average length 1.9 days). They are assumed to become infectious on the last day of the latent period, and are half as infectious as they will be after the latent period ends. After the latent period, 67% of infectives become symptomatic (state 2), and 33% are asymptomatic (state 3). These infectious states last between 3 and 6 days. Symptomatic infectives are assumed to be twice as infectious as asymptomatics, and have a chance of withdrawing home during each day of illness (see Figure 3); upon withdrawal, they only make contacts within their household and neighborhood, with transmission probabilities doubled in the household contact group, until they recover. If a school child withdraws home due to illness, one adult in the household also stays home. Each day in states 2 and 3, an infectious person has a chance to exit the state and be removed from the simulation (i.e., to recover or die — state 4). Probabilities for transition into and out of states are given in Figure 3 and are based on Longini et al. [7,16].

### Economic cost calculations

The total cost of each intervention scenario includes the cost of vaccine doses and antiviral courses used, if any; costs associated with parents staying at home with sick children and school teachers, parents, and children staying home due to school closure; costs due to illness-related absence from work; medical costs associated with illness, including outpatient visits, prescription and over-the-counter drugs, and hospitalization; and lost earnings due to death.

We use methods described by Meltzer et al. [29] to quantify most medical and work-loss costs (see also [33]). Table 5 shows the proportions of illnesses assumed to be at high risk for complications among children (0–18 years old), younger adults (19–59 years old) and seniors (over 60). Table 6 shows estimated rates of outpatient visits, hospitalizations, and death used in our calculations for children, adults, and seniors at high risk and not at high risk of complications. We chose the ‘low’ rate estimates presented in Meltzer et al. [29], which we believe to be most consistent with the relatively low R_0_ (1.4) for our model. Outpatient visit, hospitalization, and death costs are shown in Table 7; cost figures from Meltzer et al. [29] have been inflated using 2008 consumer price and medical price indexes [30-32]. All the above costs were combined with age-specific attack rates obtained from our simulation model. In addition, we assume average costs of $25 per vaccine dose or antiviral course used, consistent with previous reports [35]. Table 8 shows other costs associated with vaccination (i.e., the cost of lost time, travel, and side effects). These costs are based on [34], inflated as described above. The vaccination costs are combined with the number of used vaccination doses obtained from our simulation model. We assume that 1% of antiviral users discontinue use due to side effects; medical and other costs associated with these side effects are not included in our model.

**Table 5:** Proportions of influenza cases at high risk for complications^1^

Age Group	Proportion at High Risk
Children (0–18)	0.064
Adults (19–59)	0.144
Seniors (60+)	0.400

1. Proportions taken from Meltzer et al. [29], and adapted to our age groups

**Table 6:** Outpatient visit, hospitalization, and death rates, by age group and risk status for complications^1^

	Rates per 1000 persons ill		
	Outpatient Visits	Hospitalizations	Deaths

Not at High Risk			
Children	165	0.2	0.014
Adults	40	0.18	0.025
Seniors	45	1.5	0.28
High Risk			
Children	289	2.1	0.126
Adults	70	0.83	0.1
Seniors	79	4.0	2.76

1. Rates taken from Meltzer et al. [29]

**Table 7:** Frequency and costs (in US$) associated with influenza-related outpatient visits, hospitalizations, and deaths^1^

	Age Group		
Outcome Category Item	Children	Adults	Seniors

Outpatient Visits			
Average no. visits per case	1.52	1.52	1.52
Net payment per visit	$80.90	$62.74	$82.55
Average copayment for outpatient visit	$8.26	$6.60	$6.60
Net payment per prescription	$41.28	$59.44	$59.44
Average prescriptions per visit	0.9	1.8	1.4
Average copayment per prescription	$4.95	$4.95	$4.95
Days lost	3	2	5
Value of 1 day lost	$91.85	$141.30	$91.85
Subtotal: Per-case Outpatient Costs	$448.86	$496.50	$679.47
			
Hospitalization			
Hospital cost	$4,847.34	$9,932.42	$11,319.26
Net payment per outpatient visit	$122.17	$155.19	$168.40
Average copayment for outpatient visit	$8.26	$6.60	$6.60
Net payment for drug claims	$42.93	$69.34	$67.69
Most likely days lost	5	8	10
Value of 1 day lost	$91.85	$141.30	$91.85
Subtotal: Per-case Hospitalization Costs	$5,479.92	$11,293.96	$12,480.40
			
Deaths			
Average age (years)	9	35	74
PV earnings lost	$1,435,750	$1,466,231	$93,027
Most likely hospital costs	$5,671	$12,555	$13,718
Subtotal	$1,441,422	$1,478,788	$106,746
			
Ill but no medical care sought			
Days lost	3	2	5
Value of 1 day lost	$91.85	$141.30	$91.85
Over-the-counter drugs	$3.30	$3.30	$3.30
Subtotal: Per-case ill (no care sought)	$278.84	$285.90	$462.53

1. Estimates based on figures from Meltzer et al. [29]. Cost estimates inflated by 2008 consumer and medical price indices [30-32] as appropriate.

**Table 8:** Costs and impacts of vaccination^1^

Cost or Side Effect Item	Probability of Side Effect	Per-case Cost of Side Effect	Cost Scenario (per patient)
Assumed cost of vaccination			25.00
Patient time			5.65
Patient travel costs			5.65
Side effects			
Mild	0.0325	94	5.04
Guillain-Barré Syndrome (GBS)	0.000002	100,800	0.33
Anaphylaxis	0.000000157	2,490	0.0006

1. Estimates based on figures from Meltzer et al. [29]. Travel and side effect cost estimates inflated by 2008 consumer and medical price indices [30-32] as appropriate.

To estimate costs of ill individuals staying home and work-loss associated with parents staying at home with sick children, we multiplied the number of days (obtained from our simulation model) with the inflation-adjusted average value of lost days from Table 7. Similarly, we estimated the average number of teachers at schools and daycares by dividing the total number of such teachers in Hamilton [36] among the schools and daycares in our model. To estimate the cost of lost teacher productivity due to school closures, we multiplied the number of days schools and daycares are closed in our simulation model by the average number of teachers at Hamilton schools and daycares and by the average value of a day of lost work obtained from Table 7.

**Figure 7:**
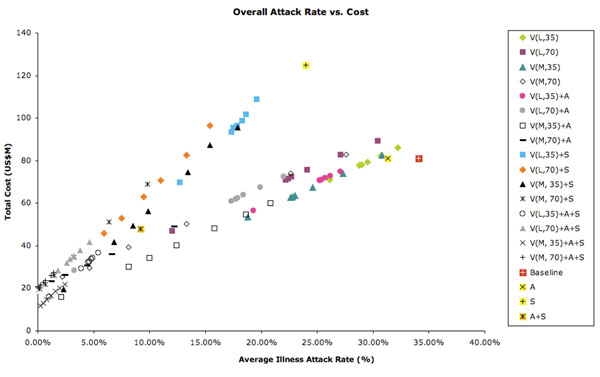
Total cost of modeled intervention strategies versus the average illness attack rate

Table S1 shows age-stratified and overall illness attack rates for all modeled scenarios, along with total cost estimates. Figure 7 depicts the total cost (US$) plotted vs. average overall illness attack rate for each intervention.

## Authors' contributions

Study conception and design: AN, SA, DG, KLT

Simulation model development: AN, WC, KLT, SA, MLL, DG, BS, DNF

Analysis and interpretation of simulation results: AN, SA, DG, WC

Drafting of manuscript: SA, AN, DG

All authors read and approved the final manuscript.

## Competing interests

DNF has received grant matching funds from Sanofi Pasteur, which manufactures a vaccine for use against influenza A (H1N1)-2009 outside Canada.

## Supplementary Material

Additional file 1 Supplementary Data for Reactive Strategies for Containing Developing Outbreaks of Pandemic Influenza Click here for file
