# Supplementary material for: Reactive strategies for containing developing outbreaks of pandemic influenza
Source: BMC Public Health. 2011 Feb 25;11(Suppl 1):S1. doi: 10.1186/1471-2458-11-S1-S1 (PMC3317583; doi:10.1186/1471-2458-11-S1-S1)
Supplement: Additional file 1 — Supplementary Data for Reactive Strategies for Containing Developing Outbreaks of Pandemic Influenza [file 1471-2458-11-S1-S1-S1.doc]

# Additional file. Supplementary Data for Reactive Strategies for Containing Developing Outbreaks of Pandemic Influenza

Sigrún Andradóttir^1^, Wenchi Chiu^1^, David M. Goldsman*^1^, Mi Lim Lee^1^, Kwok-Leung Tsui^1^, Beate Sander^2,3,4^, David N. Fisman^5^, Azhar Nizam^6^

^1^H. Milton Stewart School of Industrial and Systems Engineering, Georgia Institute of Technology, Atlanta, Georgia, USA;

^2^Toronto Health Economics and Technology Assessment Collaborative, Toronto, Ontario, Canada;

^3^Department of Health Policy, Management and Evaluation, University of Toronto, Toronto, Ontario, Canada;

^4^Division of Clinical Decision-Making and Health Care Research, University Health Network, Toronto, Ontario, Canada;

^5^Department of Epidemiology, Dalla Lana School of Public Health, University of Toronto, Toronto, Ontario, Canada;

^6^Department of Biostatistics and Bioinformatics, Emory University, Atlanta, USA.

* Corresponding author

Table S1. Average Age-stratified and Overall Illness Attack Rates (%) and Cost Estimates.

|  | Average Overall Illness Attack Rate (%) | | | | | | Cost (US$m) |
| --- | --- | --- | --- | --- | --- | --- | --- |
| Intervention | Pre-schoolers | School Children | Younger Adults | Adults | Older Adults | Overall |
| No Intervention | 29.5 | 55.9 | 40.8 | 14.3 | 11.0 | 34.1 | 81.1 |
| **Vaccination Only, Low Efficacy, 35% Coverage** | | | | | | | |
| Pre-vaccination | 21.2 | 47.9 | 30.2 | 9.5 | 7.3 | 26.1 | 71.1 |
| Reactive Vaccination^1^: |  |  |  |  |  |  |  |
| Initial Delay: None Add’l Delay: Yes | 24.0 | 51.0 | 33.8 | 11.0 | 8.5 | 28.8 | 77.7 |
| Initial Delay: 30 Day Add’l Delay: Yes | 24.6 | 51.6 | 34.6 | 11.4 | 8.8 | 29.5 | 79.3 |
| Initial Delay: 60 Day Add’l Delay: Yes | 27.4 | 54.2 | 38.3 | 13.1 | 10.1 | 32.2 | 86.0 |
| Initial Delay: None Add’l Delay: No | 24.0 | 51.0 | 33.8 | 11.0 | 8.4 | 28.8 | 77.7 |
| Initial Delay: 30 Day Add’l Delay: No | 24.2 | 51.3 | 34.0 | 11.1 | 8.5 | 29.0 | 78.1 |
| Initial Delay: 60 Day Add’l Delay: No | 25.6 | 52.6 | 36.3 | 12.2 | 9.3 | 30.7 | 82.2 |
| **Vaccination Only, Low Efficacy, 70% Coverage** | | | | | | | |
| Pre-vaccination | 8.8 | 25.3 | 13.1 | 3.6 | 2.8 | 12.0 | 47.0 |
| Reactive Vaccination: |  |  |  |  |  |  |  |
| Initial Delay: None Add’l Delay: Yes | 17.8 | 43.2 | 25.4 | 7.7 | 5.9 | 22.4 | 71.6 |
| Initial Delay: 30 Day Add’l Delay: Yes | 19.2 | 45.1 | 27.7 | 8.6 | 6.6 | 24.1 | 75.7 |
| Initial Delay: 60 Day Add’l Delay: Yes | 25.4 | 52.2 | 36.0 | 12.1 | 9.3 | 30.4 | 89.4 |
| Initial Delay: None Add’l Delay: No | 17.4 | 42.9 | 25.1 | 7.6 | 5.8 | 22.2 | 71.1 |
| Initial Delay: 30 Day Add’l Delay: No | 18.1 | 43.5 | 25.8 | 7.9 | 6.0 | 22.7 | 72.4 |
| Initial Delay: 60 Day Add’l Delay: No | 22.0 | 48.2 | 31.8 | 10.3 | 7.8 | 27.1 | 83.0 |
| **Vaccination Only, Moderate Efficacy, 35% Coverage** | | | | | | | |
| Pre-vaccination | 14.4 | 37.5 | 21.1 | 6.3 | 4.7 | 18.8 | 53.7 |
| Reactive Vaccination: |  |  |  |  |  |  |  |
| Initial Delay: None Add’l Delay: Yes | 18.0 | 43.4 | 26.0 | 8.0 | 6.1 | 22.8 | 63.1 |
| Initial Delay: 30 Day Add’l Delay: Yes | 19.6 | 45.5 | 28.4 | 8.9 | 6.8 | 24.6 | 67.5 |
| Initial Delay: 60 Day Add’l Delay: Yes | 25.7 | 52.5 | 36.5 | 12.3 | 9.4 | 30.8 | 82.5 |
| Initial Delay: None Add’l Delay: No | 18.0 | 43.2 | 25.8 | 7.9 | 6.0 | 22.6 | 62.8 |
| Initial Delay: 30 Day Add’l Delay: No | 18.3 | 43.7 | 26.4 | 8.1 | 6.2 | 23.0 | 63.7 |
| Initial Delay: 60 Day Add’l Delay: No | 22.2 | 48.3 | 32.1 | 10.3 | 8.0 | 27.3 | 74.1 |
|  | | | | | | | |
| 1. In reactive vaccination scenarios, two types of supply-chain delays that can affect vaccination programs are considered: an initial delay in program implementation of 0, 30, or 60 days; and additional delays after initiation of the program, such that vaccine doses become available in three equal batches, two weeks apart (rather than in one batch). | | | | | | | |

Table S1 (Continued).

|  | Average Overall Illness Attack Rate (%) | | | | | | Cost (US$m) |
| --- | --- | --- | --- | --- | --- | --- | --- |
| Intervention | Pre-schoolers | School Children | Younger Adults | Adults | Older Adults | Overall |
| **Vaccination Only, Moderate Efficacy, 70% Coverage** | | | | | | | |
| Pre-vaccination | 0.1 | 0.4 | 0.2 | 0.04 | 0.03 | 0.2 | 19.3 |
| Reactive Vaccination: |  |  |  |  |  |  |  |
| Initial Delay: None Add’l Delay: Yes | 3.3 | 10.1 | 5.0 | 1.3 | 1.0 | 4.6 | 29.7 |
| Initial Delay: 30 Day Add’l Delay: Yes | 9.7 | 26.8 | 15.0 | 4.3 | 3.3 | 13.3 | 50.2 |
| Initial Delay: 60 Day Add’l Delay: Yes | 22.3 | 48.3 | 32.6 | 10.6 | 8.1 | 27.6 | 83.0 |
| Initial Delay: None Add’l Delay: No | 1.5 | 4.7 | 2.3 | 0.6 | 0.5 | 2.2 | 25.6 |
| Initial Delay: 30 Day Add’l Delay: No | 5.7 | 17.1 | 8.9 | 2.4 | 1.9 | 8.1 | 39.5 |
| Initial Delay: 60 Day Add’l Delay: No | 17.5 | 40.5 | 26.7 | 8.3 | 6.3 | 22.6 | 74.0 |
| **Antivirals Only, 10% Coverage** | 26.0 | 53.0 | 37.3 | 12.7 | 9.6 | 31.3 | 75.9 |
| **School Closure/Social Distancing Only** | 22.5 | 43.6 | 27.7 | 8.6 | 6.7 | 24.0 | 125.0 |
| **Antivirals (10% Coverage) + School Closure/Social Distancing** | 7.1 | 18.8 | 10.2 | 2.8 | 2.2 | 9.2 | 48.0 |
| **Vaccination (Low Efficacy, 35% Coverage) + Antivirals (10% Coverage)** | | | | | | | |
| Pre-vaccination | 14.0 | 37.8 | 22.0 | 6.5 | 5.0 | 19.3 | 56.4 |
| Reactive Vaccination: |  |  |  |  |  |  |  |
| Initial Delay: None Add’l Delay: Yes | 19.6 | 46.9 | 29.3 | 9.2 | 7.0 | 25.3 | 70.8 |
| Initial Delay: 30 Day Add’l Delay: Yes | 20.1 | 47.4 | 29.9 | 9.4 | 7.1 | 25.7 | 71.8 |
| Initial Delay: 60 Day Add’l Delay: Yes | 21.4 | 48.8 | 31.6 | 10.1 | 7.7 | 27.1 | 75.0 |
| Initial Delay: None Add’l Delay: No | 19.5 | 46.7 | 29.2 | 9.1 | 6.9 | 25.2 | 70.6 |
| Initial Delay: 30 Day Add’l Delay: No | 19.7 | 47.0 | 29.5 | 9.2 | 7.0 | 25.4 | 71.1 |
| Initial Delay: 60 Day Add’l Delay: No | 20.6 | 47.9 | 30.5 | 9.6 | 7.3 | 26.2 | 72.9 |
| **Vaccination (Low Efficacy, 70% Coverage) + Antivirals (10% Coverage)** | | | | | | | |
| Pre-vaccination | 2.0 | 7.3 | 3.6 | 0.9 | 0.7 | 3.3 | 28.3 |
| Reactive Vaccination: |  |  |  |  |  |  |  |
| Initial Delay: None Add’l Delay: Yes | 12.6 | 35.9 | 19.8 | 5.7 | 4.4 | 17.7 | 62.0 |
| Initial Delay: 30 Day Add’l Delay: Yes | 13.1 | 37.2 | 20.7 | 6.0 | 4.6 | 18.4 | 63.9 |
| Initial Delay: 60 Day Add’l Delay: Yes | 16.5 | 42.3 | 25.2 | 7.6 | 5.8 | 22.0 | 72.4 |
| Initial Delay: None Add’l Delay: No | 12.2 | 34.9 | 19.4 | 5.6 | 4.2 | 17.3 | 61.1 |
| Initial Delay: 30 Day Add’l Delay: No | 12.6 | 36.2 | 20.0 | 5.8 | 4.4 | 17.9 | 62.5 |
| Initial Delay: 60 Day Add’l Delay: No | 14.6 | 39.6 | 22.5 | 6.6 | 5.1 | 19.9 | 67.4 |
| **Vaccination (Moderate Efficacy, 35% Coverage) + Antivirals (10% Coverage)** | | | | | | | |
| Pre-vaccination | 1.2 | 4.8 | 2.3 | 0.6 | 0.5 | 2.1 | 16.1 |
| Reactive Vaccination: |  |  |  |  |  |  |  |
| Initial Delay: None Add’l Delay: Yes | 6.2 | 21.1 | 11.1 | 3.0 | 2.3 | 10.0 | 34.3 |
| Initial Delay: 30 Day Add’l Delay: Yes | 10.9 | 32.2 | 17.8 | 5.1 | 3.9 | 15.8 | 48.2 |
| Initial Delay: 60 Day Add’l Delay: Yes | 15.4 | 40.4 | 23.8 | 7.1 | 5.4 | 20.8 | 60.1 |
| Initial Delay: None Add’l Delay: No | 4.8 | 17.4 | 9.0 | 2.4 | 1.8 | 8.1 | 30.1 |
| Initial Delay: 30 Day Add’l Delay: No | 8.2 | 25.9 | 13.9 | 3.9 | 2.9 | 12.4 | 40.2 |
| Initial Delay: 60 Day Add’l Delay: No | 13.3 | 36.9 | 21.0 | 6.1 | 4.7 | 18.6 | 54.7 |
|  | | | | | | | |

Table S1 (Continued).

|  | Average Overall Illness Attack Rate (%) | | | | | | Cost (US$m) |
| --- | --- | --- | --- | --- | --- | --- | --- |
| Intervention | Pre-schoolers | School Children | Younger Adults | Adults | Older Adults | Overall |
| **Vaccination (Moderate Efficacy, 70% Coverage) + Antivirals (10% Coverage)** | | | | | | | |
| Pre-vaccination | 0.03 | 0.14 | 0.05 | 0.01 | 0.01 | 0.05 | 20.7 |
| Reactive Vaccination: |  |  |  |  |  |  |  |
| Initial Delay: None Add’l Delay: Yes | 0.7 | 2.7 | 1.2 | 0.3 | 0.2 | 1.2 | 23.3 |
| Initial Delay: 30 Day Add’l Delay: Yes | 2.6 | 1.6 | 4.8 | 1.3 | 1.0 | 4.4 | 30.9 |
| Initial Delay: 60 Day Add’l Delay: Yes | 8.0 | 25.0 | 13.7 | 3.8 | 2.9 | 12.2 | 49.1 |
| Initial Delay: None Add’l Delay: No | 0.4 | 1.5 | 0.6 | 0.2 | 0.1 | 0.6 | 22.0 |
| Initial Delay: 30 Day Add’l Delay: No | 1.4 | 5.4 | 2.6 | 0.7 | 0.5 | 2.4 | 26.2 |
| Initial Delay: 60 Day Add’l Delay: No | 3.9 | 14.0 | 7.4 | 2.0 | 1.5 | 6.6 | 36.1 |
| **Vaccination (Low Efficacy, 35% Coverage) + School Closure/Social Distancing** | | | | | | | |
| Pre-vaccination | 10.9 | 26.3 | 13.9 | 3.9 | 3.1 | 12.7 | 69.9 |
| Reactive Vaccination: |  |  |  |  |  |  |  |
| Initial Delay: None Add’l Delay: Yes | 15.5 | 34.7 | 19.6 | 5.7 | 4.5 | 17.5 | 95.7 |
| Initial Delay: 30 Day Add’l Delay: Yes | 16.3 | 35.9 | 20.4 | 6.0 | 4.7 | 18.3 | 99.0 |
| Initial Delay: 60 Day Add’l Delay: Yes | 17.5 | 37.7 | 22.1 | 6.6 | 5.1 | 19.6 | 108.8 |
| Initial Delay: None Add’l Delay: No | 15.3 | 34.3 | 19.3 | 5.6 | 4.4 | 17.3 | 93.6 |
| Initial Delay: 30 Day Add’l Delay: No | 15.8 | 35.2 | 19.9 | 5.8 | 4.5 | 17.8 | 96.5 |
| Initial Delay: 60 Day Add’l Delay: No | 16.7 | 36.3 | 20.9 | 6.2 | 4.8 | 18.6 | 101.9 |
| **Vaccination (Low Efficacy, 70% Coverage) + School Closure/Social Distancing** | | | | | | | |
| Pre-vaccination | 0.5 | 1.6 | 0.7 | 0.2 | 0.1 | 0.7 | 22.0 |
| Reactive Vaccination: |  |  |  |  |  |  |  |
| Initial Delay: None Add’l Delay: Yes | 6.1 | 16.0 | 8.1 | 2.2 | 1.7 | 7.5 | 53.1 |
| Initial Delay: 30 Day Add’l Delay: Yes | 9.2 | 23.3 | 12.0 | 3.3 | 2.6 | 11.0 | 70.6 |
| Initial Delay: 60 Day Add’l Delay: Yes | 13.3 | 30.7 | 17.1 | 4.9 | 3.9 | 15.4 | 96.6 |
| Initial Delay: None Add’l Delay: No | 4.8 | 12.7 | 6.3 | 1.7 | 1.3 | 5.9 | 46.0 |
| Initial Delay: 30 Day Add’l Delay: No | 7.9 | 10.3 | 10.3 | 2.8 | 2.2 | 9.5 | 63.0 |
| Initial Delay: 60 Day Add’l Delay: No | 11.3 | 27.3 | 14.6 | 4.1 | 3.2 | 13.3 | 82.6 |
| **Vaccination (Moderate Efficacy, 35% Coverage) + School Closure/Social Distancing** | | | | | | | |
| Pre-vaccination | 1.8 | 5.0 | 2.4 | 0.6 | 0.5 | 2.3 | 19.6 |
| Reactive Vaccination: |  |  |  |  |  |  |  |
| Initial Delay: None Add’l Delay: Yes | 7.1 | 18.0 | 9.3 | 2.5 | 2.0 | 8.5 | 49.4 |
| Initial Delay: 30 Day Add’l Delay: Yes | 13.5 | 30.7 | 17.1 | 4.9 | 3.9 | 15.4 | 87.3 |
| Initial Delay: 60 Day Add’l Delay: Yes | 15.8 | 35.5 | 19.9 | 5.8 | 4.5 | 17.9 | 95.7 |
| Initial Delay: None Add’l Delay: No | 5.6 | 14.4 | 7.4 | 2.0 | 1.6 | 6.8 | 41.6 |
| Initial Delay: 30 Day Add’l Delay: No | 8.4 | 10.8 | 10.8 | 3.0 | 2.3 | 9.9 | 56.3 |
| Initial Delay: 60 Day Add’l Delay: No | 11.6 | 27.3 | 14.8 | 4.2 | 3.3 | 13.4 | 74.7 |
| **Vaccination (Moderate Efficacy, 70% Coverage) + School Closure/Social Distancing** | | | | | | | |
| Pre-vaccination | 0.03 | 0.1 | 0.04 | 0.01 | 0.01 | 0.04 | 19.1 |
| Reactive Vaccination: |  |  |  |  |  |  |  |
| Initial Delay: None Add’l Delay: Yes | 0.5 | 1.6 | 0.7 | 0.2 | 0.1 | 0.7 | 22.0 |
| Initial Delay: 30 Day Add’l Delay: Yes | 2.6 | 7.0 | 3.5 | 0.9 | 0.7 | 3.2 | 34.7 |
| Initial Delay: 60 Day Add’l Delay: Yes | 8.4 | 19.9 | 10.9 | 3.0 | 2.4 | 9.8 | 69.1 |
| Initial Delay: None Add’l Delay: No | 0.15 | 0.48 | 0.19 | 0.05 | 0.04 | 0.19 | 19.7 |
| Initial Delay: 30 Day Add’l Delay: No | 1.21 | 3.29 | 1.58 | 0.42 | 0.32 | 1.49 | 25.9 |
| Initial Delay: 60 Day Add’l Delay: No | 5.19 | 13.11 | 7.01 | 1.88 | 1.48 | 6.35 | 51.2 |
|  | | | | | | | |

Table S1 (Continued).

|  | Average Overall Illness Attack Rate (%) | | | | | | | Cost (US$m) |
| --- | --- | --- | --- | --- | --- | --- | --- | --- |
| Intervention | Pre-schoolers | School Children | Younger Adults | Adults | Older Adults | Overall | |
| **Vaccination (Low Efficacy, 35% Coverage) + Antivirals (10% Coverage) + School Closure/Social Distancing** | | | | | | | | |
| Pre-vaccination | 0.7 | 2.4 | 1.1 | 0.3 | 0.2 | 1.0 | 15.9 | |
| Reactive Vaccination: |  |  |  |  |  |  |  | |
| Initial Delay: None Add’l Delay: Yes | 3.1 | 9.8 | 4.8 | 1.3 | 1.0 | 4.5 | 32.2 | |
| Initial Delay: 30 Day Add’l Delay: Yes | 3.3 | 10.7 | 5.4 | 1.4 | 1.1 | 4.9 | 34.2 | |
| Initial Delay: 60 Day Add’l Delay: Yes | 3.6 | 11.7 | 5.6 | 1.5 | 1.2 | 5.4 | 36.8 | |
| Initial Delay: None Add’l Delay: No | 2.6 | 8.4 | 4.2 | 1.1 | 0.8 | 3.9 | 29.2 | |
| Initial Delay: 30 Day Add’l Delay: No | 3.1 | 10.0 | 5.0 | 1.3 | 1.0 | 4.6 | 32.6 | |
| Initial Delay: 60 Day Add’l Delay: No | 3.2 | 10.4 | 5.2 | 1.4 | 1.1 | 4.8 | 33.8 | |
| **Vaccination (Low Efficacy, 70% Coverage) + Antivirals (10% Coverage) + School Closure/Social Distancing** | | | | | | | | |
| Pre-vaccination | 0.2 | 0.5 | 0.2 | 0.1 | 0.04 | 0.2 | | 21.3 |
| Reactive Vaccination: |  |  |  |  |  |  | |  |
| Initial Delay: None Add’l Delay: Yes | 1.8 | 5.9 | 2.7 | 0.7 | 0.6 | 2.6 | | 32.0 |
| Initial Delay: 30 Day Add’l Delay: Yes | 2.3 | 7.1 | 3.4 | 0.9 | 0.7 | 3.2 | | 35.2 |
| Initial Delay: 60 Day Add’l Delay: Yes | 3.2 | 10.0 | 4.9 | 1.3 | 1.0 | 4.6 | | 41.7 |
| Initial Delay: None Add’l Delay: No | 1.2 | 3.9 | 1.9 | 0.5 | 0.4 | 1.8 | | 28.4 |
| Initial Delay: 30 Day Add’l Delay: No | 2.0 | 6.4 | 3.1 | 0.8 | 0.6 | 2.9 | | 33.6 |
| Initial Delay: 60 Day Add’l Delay: No | 2.7 | 8.4 | 4.1 | 1.1 | 0.8 | 3.8 | | 37.8 |
| **Vaccination (Moderate Efficacy, 35% Coverage) + Antivirals (10% Coverage) + School Closure/Social Distancing** | | | | | | | | |
| Pre-vaccination | 0.1 | 0.6 | 0.2 | 0.1 | 0.04 | 0.2 | | 11.9 |
| Reactive Vaccination: |  |  |  |  |  |  | |  |
| Initial Delay: None Add’l Delay: Yes | 0.5 | 1.9 | 0.9 | 0.2 | 0.2 | 0.8 | | 14.9 |
| Initial Delay: 30 Day Add’l Delay: Yes | 1.1 | 3.7 | 1.7 | 0.5 | 0.3 | 1.6 | | 18.6 |
| Initial Delay: 60 Day Add’l Delay: Yes | 1.5 | 5.3 | 2.5 | 0.6 | 0.5 | 2.4 | | 22.0 |
| Initial Delay: None Add’l Delay: No | 0.3 | 1.1 | 0.5 | 0.1 | 0.1 | 0.5 | | 13.1 |
| Initial Delay: 30 Day Add’l Delay: No | 0.8 | 2.8 | 1.3 | 0.3 | 0.3 | 1.2 | | 16.6 |
| Initial Delay: 60 Day Add’l Delay: No | 1.2 | 4.4 | 2.1 | 0.5 | 0.4 | 2.0 | | 20.2 |
| **Vaccination (Moderate Efficacy, 70% Coverage) + Antivirals (10% Coverage) + School Closure/Social Distancing** | | | | | | | | |
| Pre-vaccination | 0.02 | 0.05 | 0.02 | 0.01 | 0.00 | 0.02 | | 20.6 |
| Reactive Vaccination: |  |  |  |  |  |  | |  |
| Initial Delay: None Add’l Delay: Yes | 0.2 | 0.6 | 0.2 | 0.1 | 0.1 | 0.2 | | 21.6 |
| Initial Delay: 30 Day Add’l Delay: Yes | 0.5 | 1.6 | 0.7 | 0.2 | 0.1 | 0.7 | | 23.8 |
| Initial Delay: 60 Day Add’l Delay: Yes | 0.9 | 3.2 | 1.5 | 0.4 | 0.3 | 1.4 | | 27.4 |
| Initial Delay: None Add’l Delay: No | 0.1 | 0.3 | 0.1 | 0.03 | 0.02 | 0.1 | | 20.1 |
| Initial Delay: 30 Day Add’l Delay: No | 0.3 | 1.1 | 0.5 | 0.1 | 0.1 | 0.5 | | 22.8 |
| Initial Delay: 60 Day Add’l Delay: No | 0.8 | 2.7 | 1.3 | 0.3 | 0.3 | 1.2 | | 26.1 |
